# The role of direct oral anticoagulants in the era of COVID-19: are antiviral therapy and pharmacogenetics limiting factors?

**DOI:** 10.3325/cmj.2022.63.287

**Published:** 2022-06

**Authors:** Hrvoje Roguljić, Jerko Arambašić, Vjera Ninčević, Lucija Kuna, Igor Šesto, Ashraf Tabll, Robert Smolić, Aleksandar Včev, Dragan Primorac, George Y. Wu, Martina Smolić

**Affiliations:** 1 Department of Pharmacology, Faculty of Medicine Osijek, J. J. Strossmayer University of Osijek, Osijek, Croatia; 2 University Hospital Osijek, Osijek, Croatia; 3 Department of Pharmacology and Biochemistry, Faculty of Dental Medicine and Health Osijek, J. J. Strossmayer University of Osijek, Osijek, Croatia; 4 Department of Pathophysiology and Physiology with Immunology, Faculty of Dental Medicine and Health Osijek, Josip Juraj Strossmayer University of Osijek, Osijek, Croatia; 5 Department of Cardiology, Magdalena Clinic for Cardiovascular Diseases, Krapinske Toplice, Croatia; 6 Microbial Biotechnology Department, Genetic Engineering and Biotechnology Research Division, National Research Centre, Dokki, Egypt; 7 Department of Immunology, Egypt Center for Research and Regenerative Medicine (ECRRM), Cairo, Egypt; 8 St. Catherine Specialty Hospital, Zabok/Zagreb, Croatia; 9 Eberly College of Science, The Pennsylvania State University, University Park, State College, PA; 10 The Henry C. Lee College of Criminal Justice and Forensic Sciences, University of New Haven, West Haven, CT, USA; 11 University of Split, Medical School, Split, Croatia; 12 University of Rijeka, Medical School, Rijeka, Croatia; 13 Medical School REGIOMED, Coburg, Germany; 14 University of Mostar, Medical School, Mostar, Bosnia and Herzegovina; 15 Department of Medicine, Division of Gastroenterology-Hepatology, University of Connecticut Health Center, Farmington, CT, USA

## Abstract

In patients with COVID-19, thromboinflammation is one of the main causes of morbidity and mortality, which makes anticoagulation an integral part of treatment. However, pharmacodynamic and pharmacokinetic properties of direct oral anticoagulants (DOACs) limit the use of this class of anticoagulants in COVID-19 patients due to a significant interference with antiviral agents. DOACs use in COVID-19 hospitalized patients is currently not recommended. Furthermore, patients already on oral anticoagulant drugs should be switched to heparin at hospital admission. Nevertheless, outpatients with a confirmed diagnosis of COVID-19 are recommended to continue prior DOAC therapy. More studies are required to clarify the pathogenesis of COVID-19-induced derangement of the coagulation system in order to recommend an appropriate anticoagulant treatment.

In December 2019, severe acute respiratory syndrome coronavirus-2 (SARS-CoV-2) infection causing COVID-19 reached the epidemic proportions. Since then, the disease has spread worldwide, causing global morbidity and mortality, and has placed catastrophic burden on health care systems. This new infectious disease usually manifests with mild or no symptoms, but in some cases it can lead to a more serious clinical course due to pneumonia and acute respiratory distress syndrome (ARDS) (1). Although it primarily causes a respiratory disease, the virus affects many cell types, resulting in adverse systemic effects (2). COVID-19 patients showed an increased incidence of thromboembolic events, which brought anticoagulant drugs to the spotlight.

The number of patients using oral anticoagulants has been steadily increasing, with DOACs currently accounting for approximately 75% of oral anticoagulant prescriptions (3). During the last two decades, direct factor Xa inhibitors rivaroxaban, edoxaban, and apixaban and direct thrombin inhibitor dabigatran have been established as a standard of treatment in venous thromboembolism and atrial fibrillation. Due to their well known pharmacokinetic and pharmacodynamic characteristics, DOACs exhibit many advantages compared with low molecular weight heparin (LMWH) and vitamin K antagonists (VKA), which makes them the preferred drugs for atrial fibrillation and venous thromboembolism treatment. A relatively rapid onset of action, quick elimination pathways, and available antidotes make them convenient and desirable anticoagulant drugs (4).

Due to a widespread use of DOACs, the issue of interference between COVID-19-induced hypercoagulability and this class of anticoagulants has been raised. Furthermore, many patients on chronic DOAC treatment are hospitalized because of severe forms of COVID-19, which brings up the question of appropriate anticoagulation in hospitalized patients.

## COVID-19-induced hypercoagulability

In patients with COVID-19, thromboinflammation is a major cause of morbidity and mortality. COVID-19 causes a hypercoagulable disorder presenting as arterial and venous thrombotic incidents. Coagulopathy similar to disseminated intravascular coagulation (DIC) is present in a huge number of people hospitalized with COVID-19 (5,6). The etiology of COVID-19-associated coagulopathy is still unclear and involves many various cell types. In fact, observational research and case reports have demonstrated that some patients with COVID-19 admitted to the ICU met the International Society on Thrombosis and Hemostasis (ISTH) criteria for DIC. The most likely explanation for these differing statements is that although COVID-19-associated coagulopathy has some common pathophysiological elements with DIC, it has features of a separate entity. In COVID-19, the fluctuating condition of hypercoagulability also depends on the involvement of cells such as platelets, endothelial cells, and leukocytes and on the sampling time through the time of infection (6,7). The virus can promote the activation of the inflammatory response that includes increased inflammatory markers such as tumor necrosis factor (TNF), interferon-1 (INF-1), interleukin-6 (IL-6), and IL-12. This can result in the enlistment of immune cells, including neutrophils, leading to neutrophil extracellular trap (NET) formation. Increased levels of inflammatory markers in people with severe COVID-19 can initiate a hyperinflammatory reaction referred to as “cytokine storm,” which has been associated with poor outcomes. Furthermore, the virus may lead to the hyperactivation of platelets and endothelial injury, and the release of tissue factor, plasminogen activator inhibitor-1, and increased von-Willebrand factor, which activate the coagulation pathway (8). In patients with COVID-19, IL-6 levels correlate directly with fibrinogen levels, as well as with the increased levels of prothrombotic acute-phase reactants such as vWF, fibrinogen, and factor VIII. Prolonged immobilization also plays an important role in blood stasis, which is typical for the most serious forms of the illness (9).

Patients with COVID-19 have a higher frequency of venous thromboembolism, most likely due to severe inflammation, coagulopathy, immobilization, and initial phases of DIC (10). While some reports suggest that thrombotic incidents may have been produced by immobilization and insufficient use of thromboprophylaxis, some authors reported thrombosis even with thromboprophylaxis, proposing an explicit association between COVID-19 and thrombosis (11). Coagulopathy is one of the most significant indicators of poor outcomes in COVID-19. For example, in patients with COVID-19 pneumonia, abnormal coagulation tests were associated with a fatal outcome (12). Furthermore, patients with COVID-19 pneumonia who later died had significantly higher D-dimers, fibrin degradation products (FDP), and longer prothrombin time (PT) upon hospital admission compared with surviving patients (13). COVID-19 patients have an elevated risk of thrombosis due to impaired mobility or immobility, acute inflammatory pathophysiological events leading to hypercoagulable blood, and possibly vascular endothelial damage, which represent all three elements of Virchow's triad. The ISTH recommends the determination of D-dimer, PT, and the platelet count in all patients with COVID-19, which may help to stratify patients in need of hospitalization. It also recommends monitoring PT, D-dimers, fibrinogen, and platelets in hospitalized COVID-19 patients. If these coagulation parameters deteriorate, a more aggressive treatment is likely to be necessary (13,14).

## Interaction between DOACs metabolism and antiviral therapy

Pharmacodynamic and pharmacokinetic properties of DOACs limit the use of this class of anticoagulants in COVID-19-hospitalized patients. All DOACs are metabolized by the P-glycoprotein (P-gp) pathway, while rivaroxaban and apixaban are metabolized by the cytochrome P450 (CYP) 3A4 pathway (in a proportion of approximately 15% and 13%, respectively). Around 70% of commonly used drugs is metabolized by the CYP3A4 pathway, which makes it a critical route of drug metabolism (15). Therefore, DOACs are potentially involved in multiple drug-drug interactions with a variety of anti-COVID therapeutic drugs, which can modify their anticoagulant effects (16).

The pharmacogenetics of DOACs affect their pharmacokinetic properties. Interindividual variability regarding the efficacy and safety profile of direct anticoagulants could be related to polymorphism of the genes responsible for pharmacokinetic processes (17). To date, several single nucleotide polymorphisms (SNPs) of genes coding the proteins participating in the metabolism of DOACs have been correlated with anticoagulant treatment response. Genes with a notable effect on dabigatran pharmacokinetics are *ABCB1* gene encoding for P-gp, and *CES1* and *CES2*, liver caroboxylesterases that hydrolyze xenobiotics, since these pathways are important in the metabolism of dabigatran etexilate. P-gp pathway is also responsible for the metabolism of factor Xa inhibitors apixaban, edoxaban, and rivaroxaban, thus ABCB1 SNPs implicate alterations in the plasma levels of DOACs. Furthermore, factor Xa inhibitors are mainly metabolized by CYP-related enzymes, such as CYP3A4 and CYP2J2, making them genes of interest influencing the concentration of anticoagulants (18). However, due to the lack of sufficient evidence, pharmacogenetic testing of DOACs has still not been introduced in clinical practice, as is the case for VKA. However, a prior genotyping of patients could aid in choosing the most appropriate DOAC according to patients' individual characteristics.

Antiviral drugs have been reported to have diverse levels of success in COVID-19 treatment. Moreover, hospitalized COVID-19 patients are often treated by polypharmacy, including the use of various classes of medications, such as antiviral drugs (lopinavir/ritonavir and darunavir), antibiotics, immunosuppressive agents (tocilizumab), steroids (dexamethasone, methylprednisolone), bronchodilatators, and antihypertensives (19). Indeed, many antiviral drugs are substrates of P-gp pathway, while remdesivir and lopinavir/ritonavir are also well known CYP3A4 inhibitors (20). Testa et al (21) examined serum the levels of DOACs in hospitalized patients treated with antiviral medications such as lopinavir/ritonavir and darunavir. A cohort of 32 patients previously using DOACs were hospitalized for the treatment of COVID-19 pneumonia and 12 of them continued with DOACs regimen during hospitalization. All patients who remained on DOACs therapy had markedly increased serum levels of oral anticoagulants compared with prehospitalization levels (21). Furthermore, concomitant usage of DOACs and dexamethasone, a strong inducer of CYP3A4 and P-gp, is not recommended in patients with COVID-19-induced hypercoagulability due to potentially reduced anticoagulant effect of DOACs (22). Considering that the effect of dexamethasone lasts for around 7 days, DOACs should be continued at least one week after hospital discharge (19). Obviously, these drug-drug interactions can either enhance or reduce DOACs' anticoagulant effect, thus exposing patients to an increased risk of bleeding or thrombotic complications (23).

**Figure 1:**
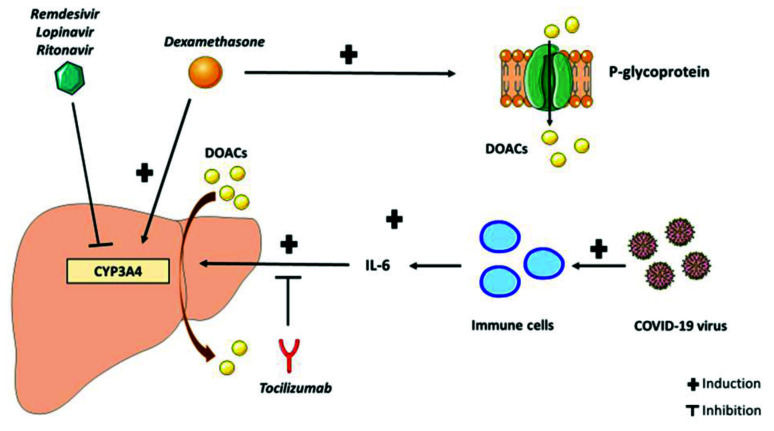
Potential interactions between COVID-19 antiviral treatment and DOACs metabolism. (Figure created with Servier Medical Art, *smart.servier.com).*

Likewise, an immune response due to COVID-19 infection should be considered during the administration of anticoagulant drugs. Generally, severe SARS-CoV-2 infection is associated with high levels of various cytokines, especially with markedly increased levels of IL-6 (24). Targeting this key mediator of inflammation represents one of the main approaches to COVID-19 treatment (25). Besides immune dysregulation, IL-6 can downregulate CYP3A4 and P-gp, which suggests that the immune response by itself is capable of modulating metabolic pathways (26). *In vitro* studies demonstrated that IL-6 significantly affected the expression of liver-enriched nuclear receptors, pregnane X receptor (PXR) and constitutive androstane receptor (CAR), which are master regulators of genes involved in the elimination of xenobiotics (27). Thus, inflammatory burden characterized by increased levels of IL-6 suppresses PXR and CAR, and consequently their target genes including *CYP3A4*. Additionally, blocking IL-6 receptor with tocilizumab or sarilumab can alter CYP3A4 enzymatic activity, altering the serum levels of DOACs (16). Taken together, many potential drug-drug interactions and metabolic changes due to acute inflammatory response (Figure 1) may lead to an unpredictable and unstable DOACs' effect.

## Are DOACs an option for anticoagulation in COVID-19 hospitalized patients?

COVID-19 increases the risk for thrombosis. As discussed above, hospitalized COVID-19 patients commonly have elevated levels of D-dimers (28). Furthermore, elevated D-dimers are the predictors of mortality, which suggests that even asymptomatic patients with significantly elevated levels should be considered for hospitalization (13). The prevalence of venous thromboembolism in non-critically ill hospitalized patients is about 2.6% (29). This number significantly increases in patients admitted to the ICU. Several studies have reported an incidence of 25%-31% for venous thromboembolism in ICUs (30,31). Another study found venous thromboembolic events, especially pulmonary thromboembolism, to be significantly more prevalent in patients with COVID-19 ARDS than in patients with ARDS of other causes, despite anticoagulant treatment (32). Based on these and other accumulating data, anticoagulation prophylaxis has become a cornerstone of COVID-19 therapy. Therefore, the question of using DOACs emerged.

One of the first relevant studies on the use of DOAC in COVID-19 was a Swedish register-based cohort study (33). This study compared the outcomes of patients with ongoing DOACs use due to nonvalvular atrial fibrillation (360 patients) and patients with known cardiovascular disease who did not use DOACs (1119 patients) before COVID-19 infection. Prior usage of oral anticoagulants did not reduce the risk of either hospitalization for acute COVID-19 or ICU admission or death due to COVID-19 (33). The study with the largest cohort of patients examining the effect and safety profile of DOACs before COVID-19 diagnosis used the data from TriNetX, a global federated health research network (34). This study included 738 423 patients, with a final sample of 26 006 patients after propensity score matching (13 003 on DOACs; 13 003 not on oral anticoagulants). This study, like the previous one, demonstrated that chronic DOAC administration before COVID-19 infection did not significantly improve clinical outcomes or rates of hospital admission in a period of one month (34). However, another study showed that patients with severe forms of COVID-19 are likely to develop a cytokine storm, which predisposes thromboembolic events and increases the already high thrombotic risk of patients taking oral anticoagulants (35).

On the other hand, some studies supported the administration of DOACs in COVID-19 patients requiring hospital treatment. One of them showed that chronic DOACs administration was independently associated with a decreased rate of death in 70 patients (>70 years) with interstitial pneumonia (36). Another cohort study from Germany showed that hospitalized COVID-19 infected patients with pre-existing therapy with VKAs or DOACs had a lower risk for extracorporeal membrane oxygenation and invasive or non-invasive ventilation (37). As there is no consensus among available studies regarding DOACs and COVID-19, further studies are warranted that would elucidate the relationship of virus infection and coagulation abnormalities.

The main evidence against DOACs benefit in COVID-19 is the finding that DOACs do not ameliorate microthrombosis in COVID-19. Some authors have proposed that hyperinflammation and coagulopathy-leukothrombosis (NETosis) are the main drivers of thrombus formation in COVID-19-infected patients. This suggests that anticoagulant doses of LMWH disrupt NET-associated thrombi while DOACs do not (38). Moreover, 71.4% of hospitalized patients who died of COVID-19 developed DIC, compared with only 0.6% of surviving patients (13). This dramatic derangement of clotting system induced by COVID-19 is not appropriate for DOAC anticoagulation, making heparins a preferred anticoagulant option. Furthermore, the anti-inflammatory properties of LMWH are beneficial in acute inflammatory conditions (39). Hospitalized patients with severe forms of COVID-19 infections are prone to develop the failure of other organ systems besides the respiratory system. For example, renal failure is common in acute COVID-19. Thus, heparins, especially unfractionated heparin, are a safer anticoagulation choice due to DOACs' renal-dependent metabolism (40). Furthermore, heparin binds to COVID-19 spike proteins and IL-6, which are elevated in COVID-19 patients (41). Considering this mechanism of action, LMWH and UFH are considered to be the best anticoagulation agents for hospitalized patients (41). According to the European Society of Cardiology, LMWH should be the first option for thromboprophylaxis in all patients who do not require hemodialysis. In patients with creatinine clearance less than 15 mL/min, the use of unfractionated heparin should be considered (42). The American College of Cardiology recommends LMWH to be considered in all hospitalized patients (both ICU and non-ICU patients) and does not recommend treatment-dose DOAC-rivaroxaban as a thromboprophylaxis strategy (43).

## Efficacy of DOACs in COVID-19 outpatients

A vast majority of patients on chronic DOAC treatment after COVID-19 are treated as outpatients. This group of patients is recommended to continue the usual oral anticoagulant regimen as long as they are well hydrated to maintain adequate renal function, and specific antiviral interfering drugs are not required (44). With exception of patients with mechanical heart valves and those with antiphospholipid syndrome, it is even recommended to switch the patients from VKA to DOACs due to restricted access to blood monitoring during lockdown periods (14). Furthermore, COVID-19 outpatients with cardiometabolic diseases treated with DOACs had a reduced risk of arterial and venous thrombotic outcomes compared with those treated with VKA (45). In addition, outpatient anticoagulation with DOACs before COVID-19 diagnosis was associated with a 43% reduced risk for hospital admission (46). Interestingly, COVID-19 outpatients on chronic oral anticoagulation (DOACs/VKA) had a lower risk of all-cause mortality compared with non-anticoagulated patients (47). However, in high-risk patients regarding age or comorbidities, a prophylactic usage of rivaroxban showed no effect on COVID-19 disease progression in patients with mild form of disease (48).

## Conclusion

COVID-19 is undoubtedly associated with increased rates of thrombotic events, mainly venous thromboembolism. These deleterious incidents have mostly occurred in hospitalized patients with severe forms of the disease (49). Thus, anticoagulation management represents one of the major therapies for the treatment of COVID-19 patients.

Only parenteral anticoagulants, LMWH or UFH, are currently recommended in COVID-19 hospitalized patients with acute disease who receive anti-viral therapy. COVID-19 antiviral medications can significantly interact with pharmacodynamic and pharmacokinetic properties of DOACs, changing their efficacy and safety profile. Furthermore, patients with severe acute COVID-19 often exhibit notable coagulation derangements, consequently demanding parenteral anticoagulation. Due to their short half-life, fewer drug interactions, and potential antiviral/anti-inflammatory effects, heparins are the standard treatment option for COVID-19-induced venous thromboembolism and antithrombotic prophylaxis in hospital-treated patients (22). The same therapeutic scheme is recommended for hospitalized patients using oral anticoagulants before hospitalization for COVID-19 treatment. On the contrary, asymptomatic COVID-19 outpatients can continue their DOACs treatment in the usual manner. Although recent meta-analyses demonstrated that chronic oral coagulation, whether it be DOAC or VKA, did not reduce the high risk of all-cause mortality in COVID-19 patients (50). Further studies are needed to clarify the mechanisms of COVID-19-induced hypercoagulability as well as to propose appropriate anticoagulant treatment options for various forms of the disease.
